# Derivation of a South African tariff for the EQ-5D-5L using a personal utility function approach

**DOI:** 10.1017/S0266462325103292

**Published:** 2025-11-21

**Authors:** Aisha Moolla, Paul Schneider, Karen Hofman, Susan J. Goldstein, Evelyn Thsehla, Simon Dixon

**Affiliations:** 1 South African Medical Research Council (SAMRC)/Wits Centre for Health Economics and Decision Science - PRICELESS, School of Public Health, University of the Witwatersrand Faculty of Health Sciences, South Africa; 2 School of Medicine and Population Health, The University of Sheffield, UK

**Keywords:** technology assessment, biomedical, quality-adjusted life-years, quality of life, cost-effectiveness analysis

## Abstract

**Objectives:**

This study’s primary objective was to test the feasibility of using the online personal utility function (OPUF) approach and develop a preliminary utility tariff for the EQ-5D-5L based on a South African community sample.

**Methods:**

The need for an ethnically and socioeconomically diverse sample was seen as essential. This led to the need for interviewer assistance during completion of the survey instrument and translation of the instrument into multiple languages. English, Zulu, Tswana, and Afrikaans were chosen to allow the vast majority of a community sample people to participate. A sample size of sixty respondents was based on a previous OPUF pilot valuation study for the EQ-5D-5L, and a pilot study of twenty respondents was undertaken using the English language version of OPUF.

**Results:**

There were sixty-one respondents in the main study with most respondent characteristics being well matched with national figures, except for language. Personal utility functions could be calculated for sixty respondents, with the mean tariff showing monotonically declining utility decrements within each dimension. An examination of individual functions showed two contrasting sets of preferences that were driven by the respondents’ rating of death. A separate subgroup analysis also showed preference heterogeneity based on the home language of the respondents.

**Conclusions:**

Our study showed that the application of the OPUF approach is possible in a socioeconomically diverse population in South Africa. The examination of individual personal utility functions shows marked heterogeneity of preferences that needs to be explored further so that the source of this can be established.

## Introduction

Economic evaluation is increasingly being used for health care commissioners to identify those services that offer the best value for money. This move is most prominent within programs that use health technology assessment (HTA) as a policy evaluation framework, for example, drug reimbursement (1). Among the different forms of economic evaluation, cost-effectiveness analysis is the most commonly adopted within the various HTA frameworks seen globally (2). Within this, the use of cost-utility analysis (CUA) has become common, although there are significant differences in the methodologies adopted by different countries and organizations (2–4).

While initially this was seen only among public health systems in high-income countries (2), this has spread to privately funded systems and to low- and middle-income countries (5;6). South Africa has recommended the use of QALYs in the evaluation of pharmaceuticals since 2012 (7), and more recently published draft HTA guidelines to inform the selection of medicines to the South African national Essential Medicines List also support the use of CUA (8). However, contrary to the published recommendations, in practice, CUA remains underutilized (9).

One problem faced by South Africa, as well as many other low- and middle-income countries in the application of CUA, is the absence of context-specific utility values, that is, values based on the preferences of their local populations. The EQ-5D-5L is the most commonly used instrument within such analyses, yet even this is limited to thirty-seven official tariffs at the time of writing (https://euroqol.org/eq-5d-instruments/eq-5d-5l-about/valuation-standard-value-sets/, accessed 19/1/24). The main reason for this is that creating a national tariff requires methodological expertise and considerable financial resources. Consequently, many countries are restricted to using values from prominent countries or their nearest geographical neighbors for which a tariff is available. In the case of South Africa, utility values for the United Kingdom (UK) and Zimbabwe have been used previously (10;11). However, the development of economic evaluation guidelines in South Africa that are heavily influenced by international bodies (7;8) has produced a growing sense of urgency for a national value set for the EQ-5D-5L.

To gain the benefits of a country-specific value set, but without the expense of a full valuation study, several countries have adopted cheaper methods to estimate their own value set. One such method, developed by Kharroubi and Rowen, adopts a Bayesian approach that uses existing data sets from other countries alongside a smaller survey to produce representative national values (12). Another is the use of an abbreviated EQ-5D-5L valuation methodology that uses composite time trade-off tasks without the associated discrete choice experiment tasks used in the full valuation method (13). While less data intensive than the full valuation, both of these methods still require sizeable surveys and analytical expertise that is not available in some countries. Another approach – using personal utility functions (PUFs) – avoids these two problems as each respondent provides extensive preference information, allowing tariffs to be estimated from much smaller samples and with reduced analytical burden (14). The efficiency of this approach is illustrated in a previous study (14), where a sample of only 50 participants yielded group tariff estimates with relatively narrow confidence intervals, indicating reasonable precision.

This study assesses the feasibility of using the personal utility function (PUF) approach to derive a preliminary, context-specific value set for the South African general population. To achieve this, we pursue five objectives:

- Identify the main requirements of a utility survey
- Adapt the PUF approach to allow its use in South Africa
- Pilot the methods in a community sample
- Apply the methods to a community sample in a full study
- Generate an initial tariff estimation

## Methods

The starting point for the estimation of a South African utility tariff was the online implementation of the PUF approach (OPUF). OPUF had already been applied to the EQ-5D-5L descriptive system as a stand-alone online survey in previous studies conducted in the UK (15) and Germany (16).

### Main requirements of a utility survey

A group of researchers comprising health economists, social scientists, and survey specialists with a knowledge of policy development and implementation in South Africa was convened to identify how OPUF should be applied locally. One of the most important requirements was considered to be that the survey sample should come from a wide range of population groups as characterized by age, gender, socioeconomic status, and ethnicity. The need for an ethnically diverse sample was seen as especially important given the multicultural nature of South Africa and its recent political history.

This requirement highlighted the need for two significant changes to the use of OPUF compared to previous studies. First, due to lower literacy rates, it was thought that interviewer assistance would need to be on-hand during completion of the survey instrument. This necessitated interviewer training and a pilot survey. Second, the survey instrument would need to be translated into languages other than English; South Africa has twelve official languages (which includes South African sign language). Translated versions of the instrument were obtained from EuroQol, which included forward/backward translation and cognitive debriefing (17). While back-translation was not feasible for the translation of the OPUF tool due to resource constraints, a two-step process of initial translation and secondary review was performed by professional translators ensuring the accuracy and cultural relevance of the translated content. Once content was translated by a first translator, a secondary translator reviewed the translated text confirming its accuracy and cultural appropriateness. Discussion between both translators took place to resolve any discrepancies.

The choice of non-English languages was inevitably a compromise between practicality/cost and comprehensiveness. Based on the 2019 South African General Household Survey data reporting “language most spoken at home,” four languages would directly cover 72.4 percent of the population; these were English (8.9 percent), Afrikaans (12.1 percent), Zulu (24.7 percent), and Sotho/Sepedi/Tswana (26.7 percent) (18). Given that knowledge of a second language is widespread in South Africa, we expected that these four languages would allow the vast majority of the sampled people to participate.

### Sample

A sample size of sixty respondents was based on a previous OPUF pilot valuation study for the EQ-5D-5L which showed that the estimation of a stable utility function is possible with that number of respondents (15). The sample was designed to reflect the overall national population in terms of age (20–39 years, 40–59 years, and 60+ years), ethnicity (Black African, Colored, Asian or Indian, and White), gender (male and female), language (English, Afrikaans, Zulu, and Sotho/Sepedi/Tswana), and wealth. All interviews were undertaken in Gauteng province (the most populous in South Africa). Quota sampling was selected to achieve a sample that met specific targets based on predetermined sample characteristics. To implement this, we relied on trained local interviewers with extensive local knowledge into the demographics within various areas of the province. All interviews were undertaken by professional interviewers. Interviewers read out each question to participants and allowed them to respond, with selected prompts included in the case of illogical responses.

### Interviews

A pilot study of twenty respondents was undertaken using the English language version of OPUF that already existed. Prior to the pilot study, a half-day interviewer training program was developed with presentations given by staff from the University of Sheffield and the University of the Witwatersrand, which included study background and a walk-through of the tool. Each interviewer was then asked to interview a volunteer within the session and then report back with reflections and questions. Due to problems identified from the analysis of the pilot study, a small number of changes to the wording of the OPUF tool were made, together with the interview procedure, after discussions with the interviewers. In addition, a second round of training was undertaken with the amended tool and interview procedure. The changes to the interview procedure required the interviewer to check that the participant understood the implication of their rating and whether they wanted to reconsider their response. For example, when more severe dimension levels were rated as better than less severe ones, the interviewer would say, “You have rated that as being better quality of life than the level above. Is that right?” If they confirm that it is correct ask, “Can you explain why that is?” The interviewer would reassure them that they are difficult questions and that they are doing well.

The final version of the instrument with interviewer prompts is shown in Supplementary File 1. The main study was undertaken in three subsequent waves, each with a target sample size of twenty individuals, to allow for the quality of the data to be checked prior to further interviews being undertaken. To minimize interviewer bias, the same trained interviewers were used across all waves of the study. All interviewers were fluent in English, and those conducting interviews in additional languages were selected based on their fluency in those languages. This approach ensured that each language group was interviewed by the same set of interviewers throughout the study.

Like the pilot, the first wave used the English language version of OPUF, while the final two waves used all four languages identified above. In Wave 1, we limited the study to English-only to prioritize operational logistics, including staff training, the feasibility of conducting in-person, doorstep interviews, and ensuring the validity of responses. Expanding to multiple languages at this stage would have introduced considerable complexity, particularly through the costs of multilingual training, potentially compromising the initial focus on foundational operational processes. The target sample characteristics in each wave is shown in Supplementary Table S1.

### OPUF

A full description of the OPUF approach is available elsewhere (15), and the full code is available in an open-access repository (19). While the theory behind the approach is beyond the scope of this paper, a short description of the routing and tasks within the tool is given here to facilitate interpretation of the results. The tool is best thought of as having five sections: an introduction to the EQ-5D-5L descriptive system, valuation of dimensions, valuation of levels, anchoring of death, and sociodemographic questions. The middle three sections are the most complex; the introduction to the descriptive system simply asks the respondents to complete the EQ-5D-5L, while the sociodemographic questions were based on the South African Census.

The valuation of dimensions involves two types of tasks. First, the respondent is asked to choose the most important dimension from the EQ-5D-5L descriptive system: mobility, self-care, usual activities, anxiety/depression, or pain/discomfort. Second, using the dimension chosen as the most important as a “measuring stick,” the respondent is asked to rate the importance of the four remaining dimensions. The measuring stick is in the form of a visual analog scale, with 100 denoting that the dimension is as important as the most important one and zero denoting that the dimension is not important at all.

The valuation of the levels involves using a separate visual analog scale for each dimension, then rating Levels 2, 3, and 4 of each dimension on that scale. Each scale is bounded by “no problems” (which is automatically assigned as 100) and “extreme problems” (which is automatically assigned as zero), which correspond to Levels 1 and 5 of the EQ-5D-5L descriptive system, respectively.

Anchoring of death requires two tasks within the OPUF approach. First, a respondent must choose whether death is preferred to the worst possible health state as defined by the classification system (which is sometimes referred to as the “pits state,” or in the parlance of the EQ-5D-5L, “55555”). The least preferred of those two options is then set to zero on a visual analog scale, which has “no health problems” assigned as 100.

### Analysis

The results of the pilot and main study were analyzed qualitatively by looking at rates of illogical answers, for example, rating “slight problems” as worse than “moderate problems” in the scaling task. Preference elicitation data were then analyzed to produce mean dimension weights, level ratings, and a group tariff. Descriptive statistics were produced relating to sample composition, duration of the survey, numbers of respondents requiring assistance from the interviewer, and any additional comments given by respondents. Survey duration was calculated from the electronic timestamps of the first and last answers, while the amount of assistance required was assessed by the interviewer using a three-point scale: no/minimal assistance, some assistance, or a lot of assistance. To assess the potential influence of completion speed on data quality, an analysis was conducted comparing the rate of illogical responses between participants above (“faster responses”) and below (“slow responses”) the median completion time using a Wilcoxon rank-sum test.

For the overall study sample, an exploratory subgroup analysis was planned based on the language that the respondent uses at home, to assess heterogeneity of preferences within the population. This grouping variable was used as a proxy for cultural differences between respondents, although this is recognized as being very simplistic.

### Permissions

Permissions were granted by The EuroQoL Group to incorporate the EQ-5D-5L instrument within another valuation tool and for use of the different language versions (registration numbers 52769 and 53182). Research ethics was granted by the Human Research Ethics Committee (Medical) at the University of the Witwatersrand (M220939). The authors assert that all procedures contributing to this work comply with the ethical standards of the relevant national and institutional committees on human experimentation and with the Helsinki Declaration of 1975, as revised in 2013.

## Results

### Sample

In the pilot of twenty participants, fourteen (70 percent) respondents gave “illogical” responses in the level rating task, in that the ranking of levels implied by the descriptors (e.g., slight versus moderate) was not reflected in the ratings. Ten (50 percent) gave illogical responses in all five dimensions. It was also noted that 30 percent of the recorded level ratings had a value of either 100 or zero, which was much higher than in previous studies.

Following wording changes and further interviewer training, these rates were reduced considerably in Wave 1 of the main study, with the rest of the survey carrying on with those same methods. One further interim analysis undertaken for quality assurance identified that almost all remaining illogical responses were made in the presence of one specific interviewer and so this interviewer was removed from the interviewer pool. This was likely due to the interviewer being new to the role. All other interviewers were highly experienced and performed the rest of the interviews. Further details of illogical and extreme responses produced during data collection are given in Supplementary Table S2.

Across the three waves of the main survey, there were sixty-one respondents. Most respondent characteristics were well matched with national figures (<3 percentage points different), except for language (Supplementary Table S1). In summary, 52.5 percent of the sample were aged under 40 years (compared to 54.1 percent in the general adult population), 47.5 percent were female (51.1 percent general population), 75.4 percent were Black African (78.2 percent general population), and 26.2 percent were from a low-income area (33.3 percent general population). The biggest discrepancy was in relation to language most spoken at home with 45.9 percent in the sample speaking English compared to 11.1 percent in the sample. This discrepancy was due to the decision to undertake the Wave 1 interviews solely using an English-language version of the tool. This Wave contributed twenty respondents to the full sample of sixty-one. Overall, the survey was made available in four languages: six respondents took the Afrikaans version, twenty-eight in English, fifteen in Tswana, and twelve in Zulu.

On average, it took respondents 11 minutes to complete the survey, ranging from 3 to 31 minutes. There was no statistically significant difference in the rate of illogical responses between “fast” and “slow” responses (*p* = 0.67). A total of thirty-one (51 percent) respondents required no or only minimal assistance from the interviewer, twenty-one (34 percent) required some, and eight (13 percent) required a lot of assistance. For the derivation of individual and full-sample tariffs, one respondent was removed from the analysis because they set “dead” as being equal to full health in the anchoring task, which makes it impossible to derive anchored coefficients or to construct a PUF (because of division by zero).

### Dimension weighting and level rating

**Figure 1.**
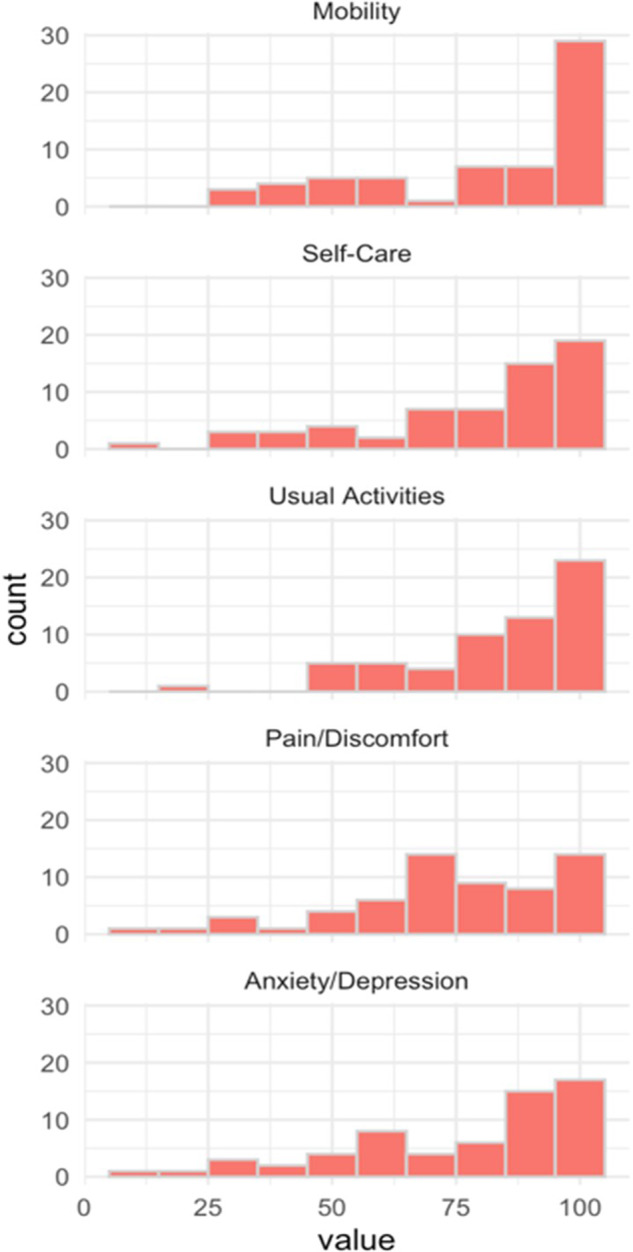
Dimension weights.

The distribution of responses for the rating of each dimension is shown in Figure 1. The median values for mobility, self-care, usual activities, pain/discomfort, and anxiety/depression were 92, 88, 88, 76, and 89, respectively. The mean values for the ratings of the three intermediate levels (“slight,” “moderate,” and “severe”) are shown in Table 1.

**Table 1.** Level ratings (*n* = 60)

	Mobility	Self-care	Usual activities	Pain/discomfort	Anxiety/depression
	Mean (SD)				
Level 2 (“slight problems”)	82.4 (16)	78.9 (20)	79 (17.7)	79.6 (17.2)	79.7 (16.9)
Level 3 (“moderate problems”)	63.1 (16.5)	59.5 (17.6)	62.1 (18.2)	60.6 (17)	62.2 (19.5)
Level 4 (“severe problems”)	41.8 (24.5)	39.3 (24.9)	41.8 (26.5)	41.4 (25.1)	40.6 (26.2)

SD, standard deviation.

### Anchoring task

**Figure 2.**
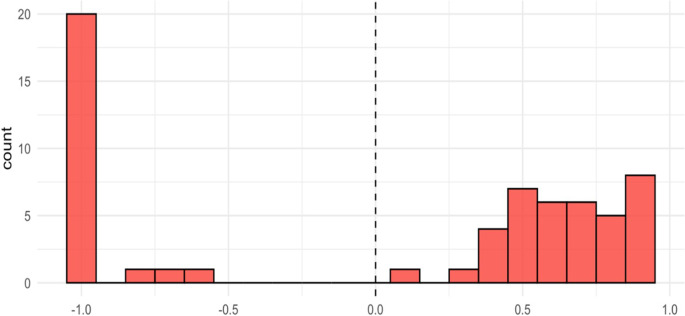
Implied valuations of health state 55555.

A total of twenty-three (38 percent) respondents indicated that they preferred dead over EQ-5D-5L health state 55555, and conversely, thirty-eight (62 percent) preferred state 55555 over dead (or were indifferent). The average implied utility of state 55555 on a scale anchored on one (“no problems”) and zero (“death”) was −1.6. In line with previous unbounded approaches, the lowest anchor point was then censored at a value of minus one (20), which produced an average utility for state 555555 of 0.05. The distribution of values for “555555” showed a marked gap between the positive (from respondents who preferred “555555” over dead) and negative utility values (from respondents who preferred dead over “555555”), as shown in Figure 2.

When the dimension and level ratings are combined and transformed onto the anchored utility values, the mean decrements from Level 1 (“no problems”) for each dimension are calculated to produce a mean utility tariff (Table 2). The decrements exhibit monotonicity and, in general, are evenly spaced except for the larger decrements seen for most dimensions when moving from Level 4 to Level 5.

**Table 2.** Estimated utility decrements relative to Level 1 for the full sample

	Mobility	Self-care	Usual activities	Pain/discomfort	Anxiety/depression
	Mean (95% CI, bootstrapped using 10,000 iterations)				
Level 2	0.031 (0.021; 0.043)	0.041 (0.026; 0.059)	0.045 (0.028; 0.067)	0.035 (0.023; 0.049)	0.043 (0.022; 0.069)
Level 3	0.082 (0.059; 0.107)	0.085 (0.063; 0.109)	0.093 (0.064; 0.126)	0.076 (0.055; 0.101)	0.082 (0.054; 0.116)
Level 4	0.133 (0.099; 0.170)	0.131 (0.097; 0.167)	0.145 (0.106; 0.189)	0.118 (0.087; 0.151)	0.125 (0.089; 0.166)
Level 5	0.199 (0.153; 0.247)	0.188 (0.145; 0.234)	0.211 (0.161; 0.265)	0.173 (0.135; 0.214)	0.182 (0.137; 0.234)

CI, confidence interval.

**Figure 3.**
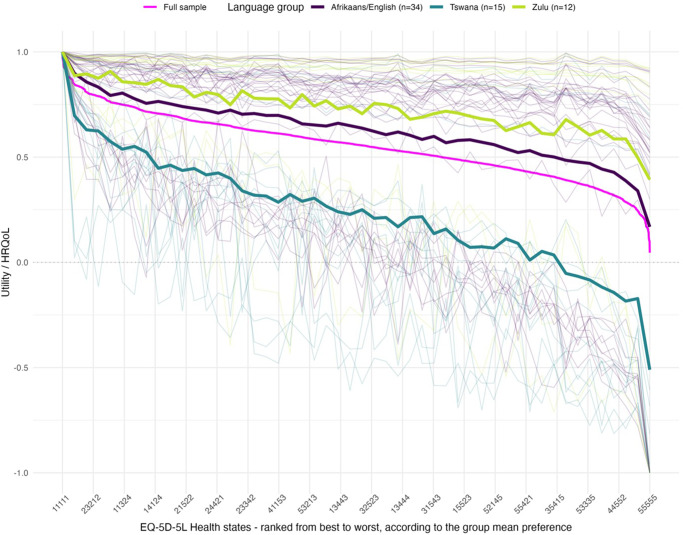
Individual, full-sample, and subgroup utility functions. HRQoL, health-related quality of life. *Note*: Simplified illustration of the group utility function (thick line) and the personal utility functions of all respondents. The colors of the individual PUF lines indicate their Euclidean distance from the average preference.

The personal utility function of each respondent can also be estimated, and a simplified illustration of the variation between these is shown in Figure 3. The values shown by the thin lines are those for each respondent for a sample of 100 health states, ranked from the best on the left to the worst on the right (according to the aggregate group preference). This shows two large groups of individuals with utility functions that are either “compressed” into the range 0.5–1.0 (those above the aggregate group preference shown by the pink line) or “stretched” over a range of 0.5 to −1.0 (those below the aggregate group preference shown by the pink line). A single respondent has a utility function that sits between these two groups, and coincidentally, the mean group function matches that individual closely.

A subgroup analysis was undertaken to examine possible preference heterogeneity. We focused our attention on the impact of possible cultural factors on preferences and used language most spoken at home, and which OPUF was made available in, as a crude indicator of this. We defined three groups by this – Tswana, Zulu, and Afrikaans/English – with the mean utility functions for these being shown in Figure 3. This shows that respondents who spoke Tswana as their main language at home had a noticeably different set of utility values.

## Discussion

### Summary

Our study showed that the application of the OPUF approach is possible in a socioeconomically diverse population in South Africa. Interviewer support was considered essential to help with completion of the OPUF tool, and even then, piloting and ongoing quality assurance was needed. All participants completed all the tasks, and a tariff could be calculated for sixty out of sixty-one respondents. Examination of the individual personal utility functions suggests that there are two different latent groups characterized by their attitudes toward death (highlighted in Figures 2 and 3), which needs further investigation.

### Strengths

The study is based on a validated preference elicitation approach, which was piloted to ensure its successful implementation in a diverse sample and with interviewer support. The ability of OPUF to generate PUFs also highlights marked differences within the sample in regard to how individuals rate full health, death, and “worst possible health” (“55555”).

### Weaknesses

The pilot identified high rates of illogical and extreme responses; however, following further training and the exclusion of one interviewer, the quality of responses was improved. Despite this, some illogical responses remained, but these responses were not excluded after discussions with interviewers as they were considered to be caused by respondents’ interpretation of the EQ-5D-5L descriptive system, rather than their understanding of the valuation task. The remaining extreme responses were not excluded as it was not possible to categorically determine whether they were the result of misunderstandings, or not.

The sample size was based on previous studies undertaken in the UK and Germany, which were shown to produce reasonably homogenous PUFs. This study, however, generated a more heterogenous set of utility functions, which means that there is higher uncertainty around the mean coefficient estimates. The main reason for this is thought to be the diversity of the South African population; however, we cannot rule out that the performance of the elicitation tool in this sample may have contributed to this as well.

Moreover, the visual inspection of the PUFs revealed that there seem to be two distinct preference groups: one group with “compressed” utilities, who indicated that they would clearly prefer the worst health state (“55555”) over being dead, and another group with “stretched” utilities, who clearly preferred being dead. This pattern was not found in any of the previous OPUF valuation studies, conducted in European countries, and may reflect differences in preferences or other factors (e.g., use of heuristics or misunderstandings). Due to the small sample sizes, meaningful statistical analyses of the composition of these two groups were not feasible.

Overall, with an average of 0.05, the utility of the worst health state observed in our study was higher than in any other OPUF or conventional EQ-5D-5L valuation studies (21). This may, in part, be explained by the high proportion of participants (62 percent), who indicated that no health state would be worse than dead and who assigned unusually high values to state “55555.”

Exploratory analysis of preference heterogeneity among population groups showed differences between some subgroups as defined by language most spoken at home. These differences were not tested for statistical significance and are based on small sample sizes; as such, the differences need to be treated with caution. It is worth recognizing that the subgroups received different versions of OPUF based on their language: Tswana, Zulu, English, and Afrikaans (with respondents in the final two languages being combined for the purposes of the subgroup analysis to overcome the small sample sizes for the two groups individually). As such, the differences may be attributable to differences in languages/translations, rather than differences in the preferences of the respondents for whom those were their primary language. However, the main determinant of the difference in preference functions appears to be due to the relative valuation of death and “55555,” which could be considered to be less susceptible to nuances of language/translation.

### Future research

The reduced sample sizes required for the application of the PUF methodology is an advantage in comparison to more common methods (e.g., TTO or DCE). However, another important advantage is that it avoids the need for statistical estimation of a utility tariff; instead, the tariff is calculated directly from the means of the individual utility functions. The statistical estimation required by the other approaches introduces uncertainty regarding the validity of the methods used, most notably, the functional form of the regression equation; any given data set for the EQ-5D, for example, can produce many different utility tariffs. This has been most exemplified by the controversy relating to the UK EQ-5D-5L tariff (22–24).

However, it is important to recognize that this additional advantage is achieved through the implicit imposition of assumptions relating to the functional form of individual preferences. In addition, the PUF methodology as developed to date does not use choice-based methods whereby different aspects of health are traded-off against “full health” or “death.” In normative terms, this is considered to be a disadvantage by some, but not all (25). Given the potential scale of health losses from adopting a utility tariff that is “wrong,” further research examining the relative merits of the PUF methodology relative to traditional methods is essential.

Identifying the cause of the heterogeneous preference identified by this study appears to be the most important priority for future research. If this is produced by genuinely different preferences, then a better characterization of the characteristics that drive this should be identifiable. Whether and how decision makers then use the mean population would then become a subsidiary issue. It is also possible that the preferences of a diverse population are more heterogeneous and that a larger sample size will smooth out the differences between the two latent groups observed in this study. Alternatively, if the heterogeneity is produced by some respondents’ inability to undertake the tasks in the desired manner, then the validity of the results needs to be called into question.

More generally, it is noted that the increased use of PUFs increases researcher’s interests in preference heterogeneity and subgroup analyses. However, insufficient thought has been given to how such analyses should be used by decision makers, for example, should funding decisions be made using the mean or using the subgroups means? The practicalities and consequences of this require in-depth consideration.

More research is also needed to further validate our valuation approach. This should include an assessment of the impact of interviewer and language effects and qualitative research to explore the cognitive processes underlying respondents. A direct comparison with EQ-5D-5L tariffs from other countries may seem interesting but would be of limited value because South Africa lacks an alternative local tariff, and preferences for health states are highly context-specific, varying due to cultural, economic, and social factors. Additionally, differences in valuation methods can lead to inconsistent utility values, complicating interpretation.

Further, there was a relatively wide range in survey completion times, from 3 to 31 minutes, with a very short minimum completion time identified. This may suggest that some participants were not fully engaged with the interview and that their responses do not accurately reflect their real preferences. However, this minimum time is consistent with another preference elicitation study that uses the OPUF approach (15), and the rate of illogical responses did not differ between “faster” and “slower” respondents, which may indicate that this is a feature of the method’s efficiency. Strategies for exploring this should be considered in futures studies.

## Conclusions

Our study shows that the OPUF approach using a small sample can be successfully implemented in a resource-constrained, socioeconomically diverse population by providing interview support. This has led to the development of a preliminary EQ-5D-5L tariff for South Africa using a sample that reflects important features of the national population. The examination of individual PUFs shows marked heterogeneity of preferences that needs to be explored further so that the source of this can be established.

## Supporting information

Moolla et al. supplementary material Moolla et al. supplementary material

## Supplementary material

The supplementary material for this article can be found at http://doi.org/10.1017/S0266462325103292.

## References

[1] Drummond M. Twenty years of using economic evaluations for drug reimbursement decisions: What has been achieved? J Health Polit Polic. 2013;38:1081–1102. 10.1215/03616878-2373148 23974475

[2] Barnieh L, Manns B, Harris A, et al. A synthesis of drug reimbursement decision-making processes in organisation for economic co-operation and development countries. Value Health. 2014;17:98–108. 24438723 10.1016/j.jval.2013.10.008

[3] Mathes T, Jacobs E, Morfeld JC, Pieper D. Methods of international health technology assessment agencies for economic evaluations-A comparative analysis. BMC Health Serv Res. 2013;13:371 24079858 10.1186/1472-6963-13-371 PMC3849629

[4] Daccache C, Karam R, Rizk R, Evers SM, Hiligsmann M. The development process of economic evaluation guidelines in low- and middle-income countries: A systematic review. Int J Technol Assess Health Care. 2022;38:e35. 35451358 10.1017/S0266462322000186

[5] Chambers JD, Enright DE, Panzer AD, et al. Examining US commercial health plans’ use of the Institute for Clinical and Economic Review’s reports in specialty drug coverage decisions. J Manag Care Spec Pharm. 2023;29:257–264. 36840954 10.18553/jmcp.2023.29.3.257 PMC10387943

[6] Falkowski A, Ciminata G, Manca F, et al. How least developed to lower-middle income countries use health technology assessment: A scoping review. Pathog Glob Health. 2023;117:104–119. 35950264 10.1080/20477724.2022.2106108 PMC9970250

[7] South African Department of Health. Guidelines for pharmacoeconomic submissions. Pretoria: National Department of Health; 2012.

[8] South African Department of Health. Health technology assessment methods guide to inform the selection of medicines to the South African National Essential Medicines List. Pretoria: National Department of Health; 2021.

[9] Sharma D, Aggarwal AK, Wilkinson T, et al. Adherence to country-specific guidelines among economic evaluations undertaken in three high-income and middle-income countries: A systematic review. Int J Technol Assess Health Care. 2021;37:e73. 34193325 10.1017/S0266462321000404

[10] Wang L, Dowdy DW, Comins CA, et al. Health-related quality of life of female sex workers living with HIV in South Africa: A cross-sectional study. J Int AIDS Soc. 2022;25:e25884. 35212470 10.1002/jia2.25884 PMC8874880

[11] Kastien-Hilka T, Rosenkranz B, Sinanovic E, Bennett B, Schwenkglenks M. Health-related quality of life in South African patients with pulmonary tuberculosis. PLoS One. 2017;12:e0174605. 28426759 10.1371/journal.pone.0174605 PMC5398494

[12] Kharroubi SA, Rowen D. Valuation of preference-based measures: Can existing preference data be used to select a smaller sample of health states? Eur J Health Econ. 2019;20:245–255. 29980950 10.1007/s10198-018-0991-1

[13] Yang F, Katumba KR, Roudijk B, et al. Developing the EQ-5D-5L value set for Uganda using the ’lite’ protocol. PharmacoEconomics. 2022;40:309–321. 34841471 10.1007/s40273-021-01101-x PMC8627844

[14] Devlin NJ, Shah KK, Mulhern BJ, Pantiri K, van Hout B. A new method for valuing health: Directly eliciting personal utility functions. Eur J Health Econ. 2019;20:257–270. 30030647 10.1007/s10198-018-0993-z PMC6438932

[15] Schneider PP, van Hout B, Heisen M, Brazier J, Devlin N. The online elicitation of personal utility functions (OPUF) tool: A new method for valuing health states. Wellcome Open Res. 2022;7:14. 36060298 10.12688/wellcomeopenres.17518.1 PMC9396078

[16] Schneider P, Blankart K, Brazier J, van Hout B, Devlin N. Using the online elicitation of personal utility functions (OPUF) approach to derive a patient-based EQ-5D-5L value set: A study in 122 patients with rheumatic diseases from Germany. Value Health. 2024;27(3):376–382. 38154596 10.1016/j.jval.2023.12.009

[17] EuroQol. Translation process [Internet]. EuroQol; 2024 [cited 2024 Nov 10]. Available from: https://euroqol.org/register/quality/version-management-committee/translation-process/

[18] Statistics South Africa. General household survey 2018. Pretoria: Statistics South Africa; 2019.

[19] Schneider P. bitowaqr/opuf_demo: OPUF zenodo version 1. 2021. 10.5281/zenodo.5773915.

[20] Dolan P. Modeling valuations for EuroQol health states. Med Care. 1997;35:1095–1108. 9366889 10.1097/00005650-199711000-00002

[21] Roudijk B, Janssen B, Olsen JA. How do EQ-5D-5L value sets differ? In: Devlin N, Roudijk B, Ludwig K, editors. Value sets for EQ-5D-5L: A compendium, comparative review & user guide. Cham (CH): Springer; 2022. 36810025

[22] Devlin NJ, Shah KK, Feng Y, Mulhern B, van Hout B. Valuing health-related quality of life: An EQ-5D-5L value set for England. Health Econ. 2018;27:7–22. 28833869 10.1002/hec.3564 PMC6680214

[23] van Hout B, Mulhern B, Feng Y, Shah K, Devlin N. The EQ-5D-5L value set for England: Response to the "quality assurance. Value Health. 2020;23:649–655. 32389231 10.1016/j.jval.2019.10.013

[24] Hernandez Alava M, Pudney S, Wailoo A. The EQ-5D-5L value set for England: Findings of a quality assurance program. Value Health. 2020;23:642–648. 32389230 10.1016/j.jval.2019.10.017

[25] Parkin D, Devlin N. Is there a case for using visual analogue scale valuations in cost-utility analysis? Health Econ. 2006;15:653–664. 16498700 10.1002/hec.1086

