# Supplementary material for: Derivation of a South African tariff for the EQ-5D-5L using a personal utility function approach
Source: Int J Technol Assess Health Care. 2025 Nov 21;41(1):e82. doi: 10.1017/S0266462325103292 (PMC12689237; doi:10.1017/S0266462325103292)
Supplement: Moolla et al. supplementary material [file S0266462325103292sup001.docx]

**Supplementary File 1.** Outline of the OPUF tool

Notes:

1. Welcome screen and consent screen have been omitted for brevity.
2. EQ-5D-5L follows the consent screen, and has been omitted for brevity.
3. The content of some screens is dynamic, i.e. it depends on previous responses (which have to be given to allow for the participant to continue).
4. Some of the pages include further explanation which are accessed via the “>Show details” hypertext links. These have been omitted for brevity.
5. Some pages include dynamic prompts that appear when anomalous responses are given. These have been omitted for brevity.

Page 1


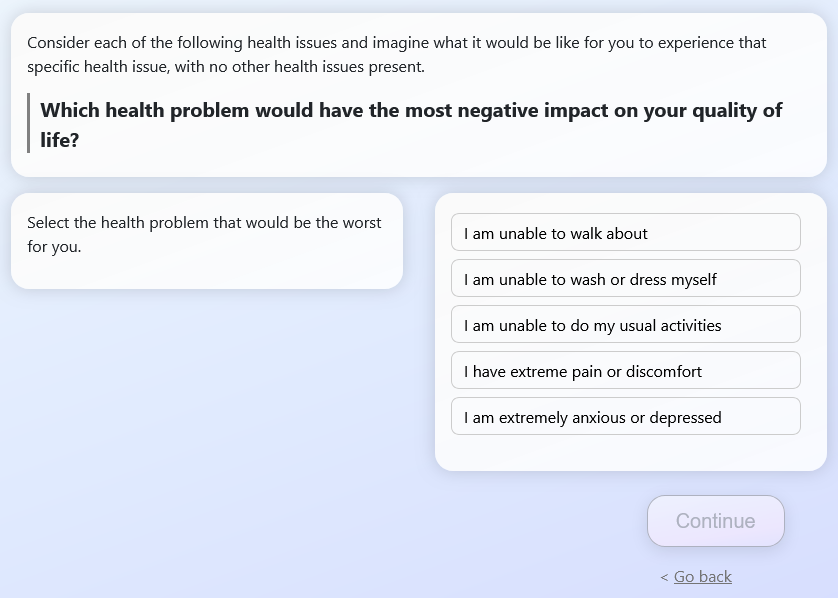


Page 2


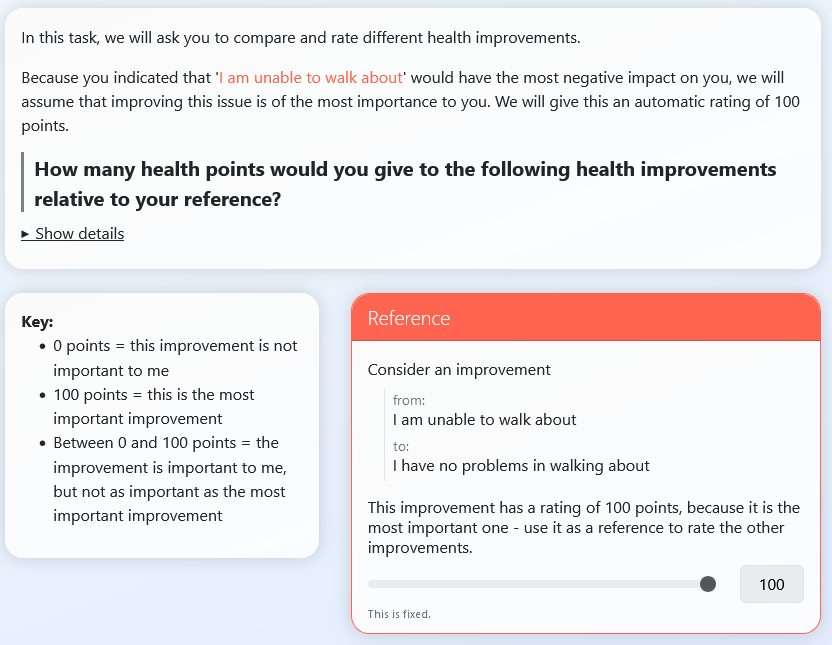

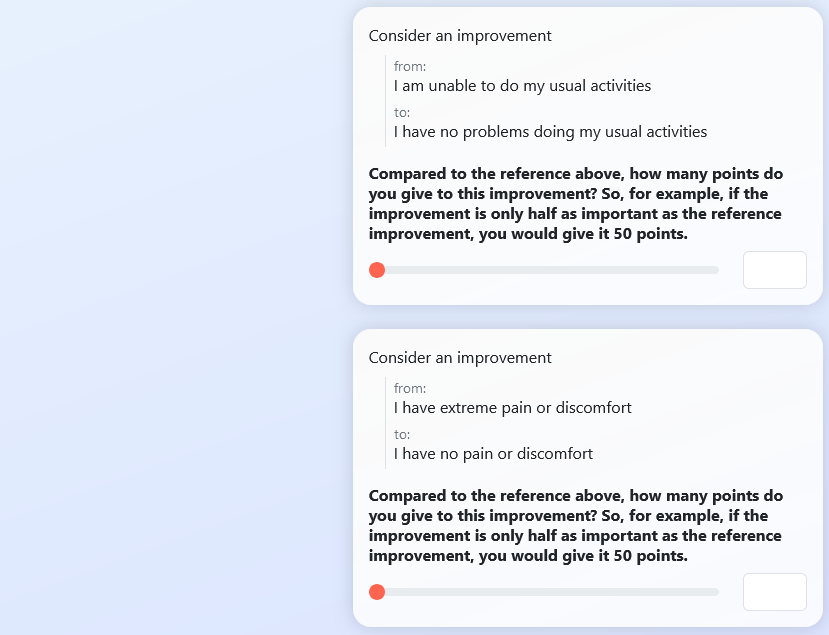

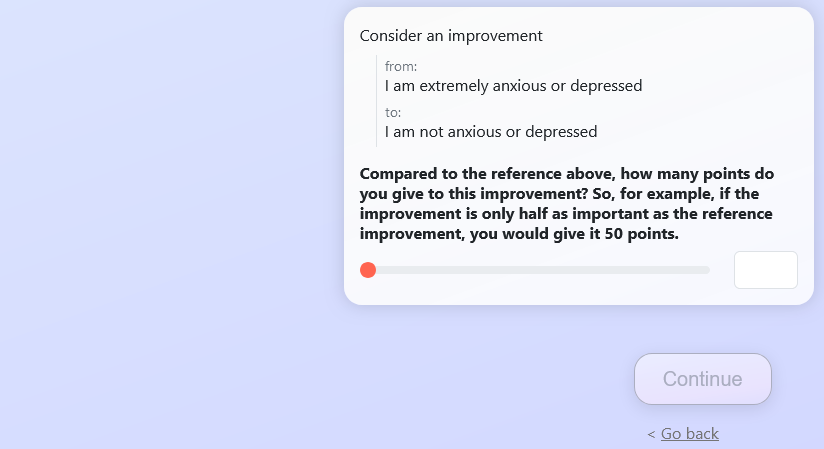


Page 3


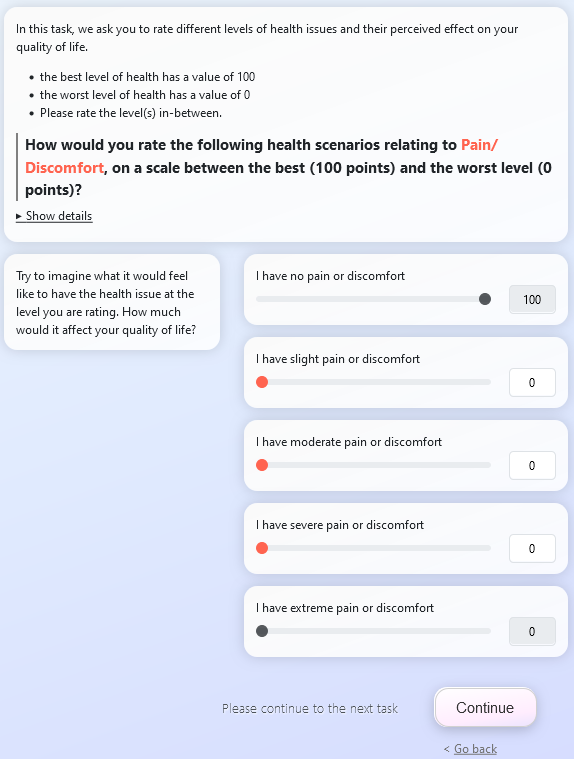


Page 4


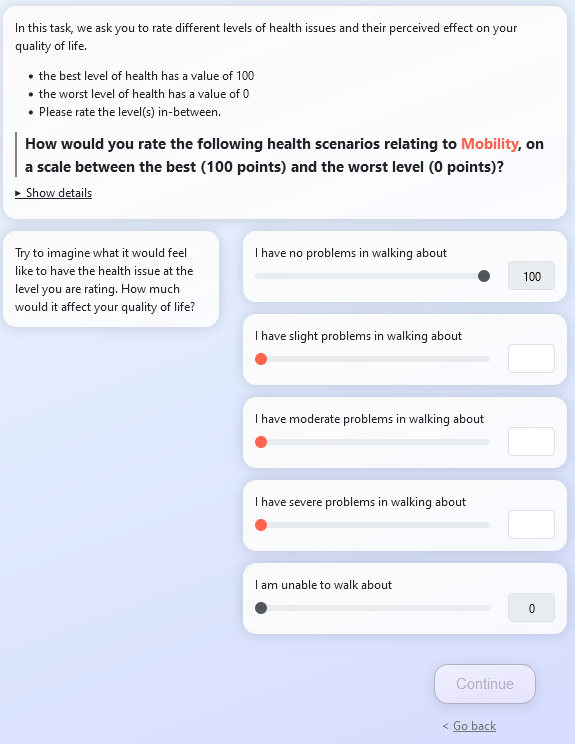


Page 5


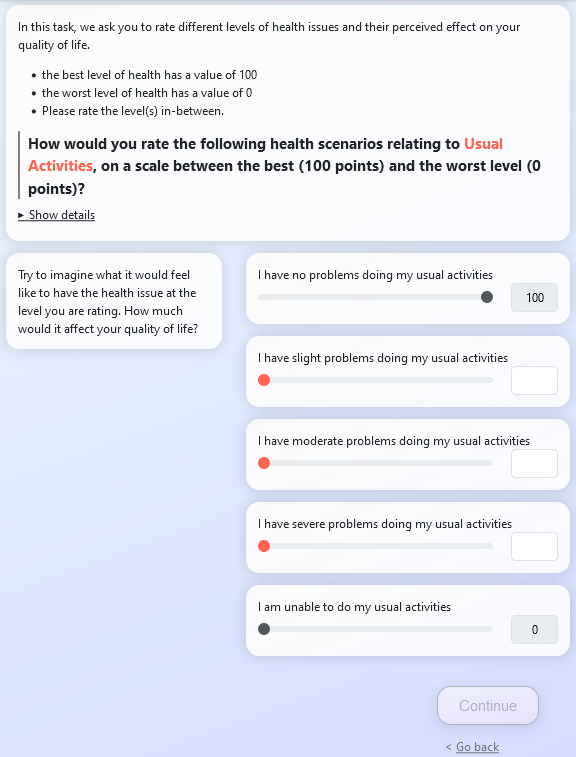


Page 6


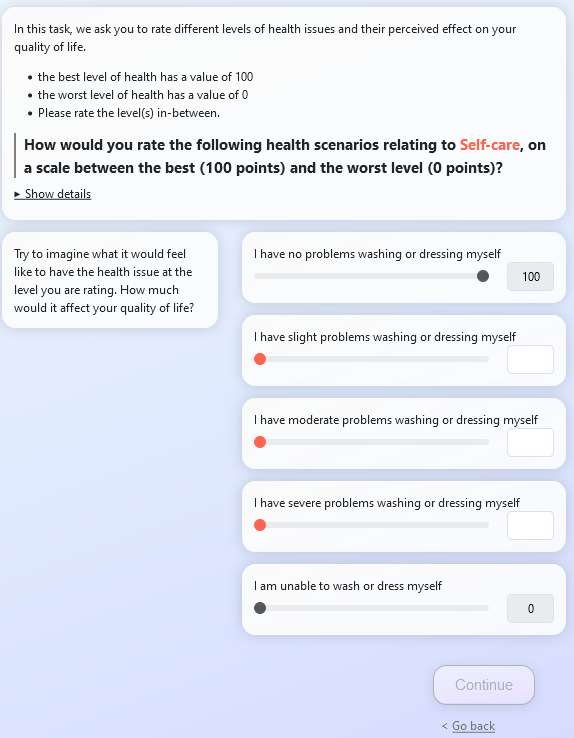


Page 7


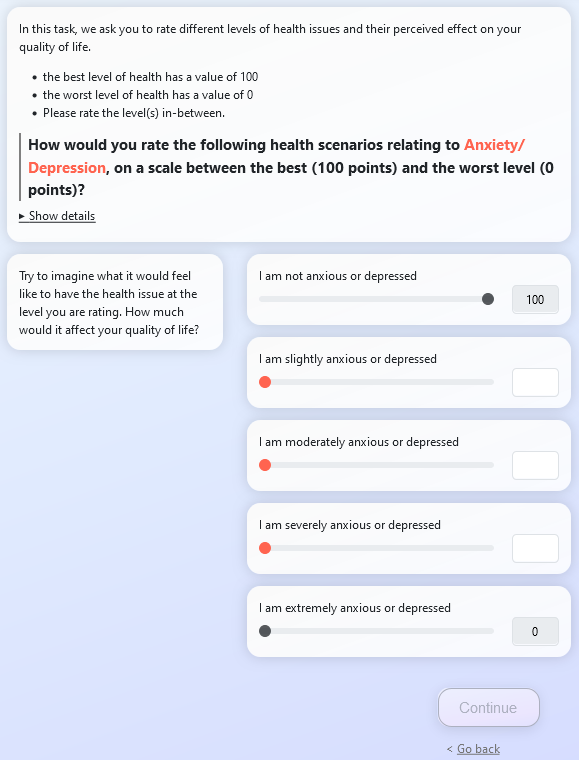


Page 8


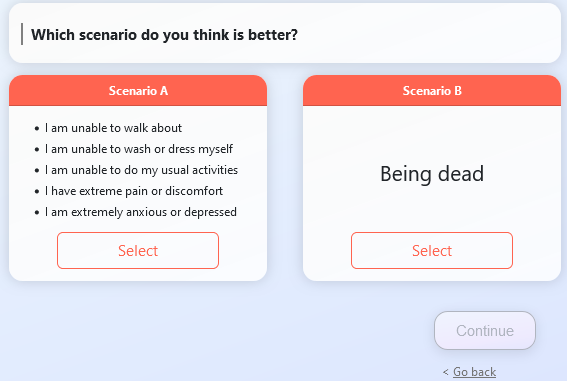


Page 9


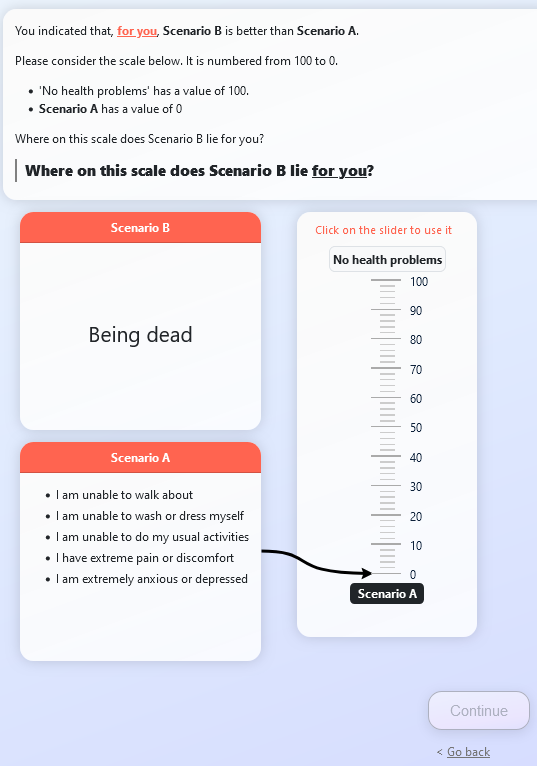


Further notes

1. Screens relating to participant demographics, participant feedback and interviewer details have been omitted for brevity.

**Supplementary Table 1.** Target characteristics by wave of data collection and sample characteristics

|  | **Wave 1 target (N=20)** | **Wave 2 target (N=20)** | **Wave 3 target (N=20)** | **Overall target (N=60)** | **Sample characteristics (N=61)** |
| --- | --- | --- | --- | --- | --- |
| **Age group (n, %)**  20-39 years  40-59 years  60+ years | 11 (55.0%)  7 (35.0%)  2 (10.0%) | 11 (55.0%)  6 (30.0%)  3 (15.0%) | 11 (55.0%)  6 (30.0%)  3 (15.0%) | 33 (55.0%)  19 (31.7%)  8 (13.3%) | 32 (52.5%)  21 (34.4%)  8 (13.1%) |
| **Gender (n, %)**  Male  Female | 10 (50.0%)  10 (50.0%) | 10 (50.0%)  10 (50.0%) | 10 (50.0%)  10 (50.0%) | 30 (50.0%)  30 (50.0%) | 32 (52.5%)  29 (47.5%) |
| **Race/ethnicity (n, %)**  Black  Coloured  Indian  White  Preferred not to say | 15 (75.0%)  2 (10.0%)  0 (0%)  3 (15.0%) | 16 (80.0%)  2 (10.0%)  1 (5.0%)  1 (5.0%) | 16 (80.0%)  2 (10.0%)  0 (0.0%)  2 (10.0%) | 47 (78.3%)  6 (10.0%)  1 (1.7%)  6 (10.0%) | 46 (75.4%)  6 (9.8%)  1 (1.6%)  7 (11.5%)  1 (1.6%) |
| **Wealth indicator (n, %)**  High income  Middle income  Low income  Not stated | 7 (35.0%)  6 (30.0%)  7 (35.0%) | 7 (35.0%)  6 (30.0%)  7 (35.0%) | 7 (35.0%)  7 (35.0%)  6 (30.0%) | 21 (35.0%)  19 (31.7%)  20 (33.3%) | 20 (32.8%)  14 (23.0%)  16 (26.2%)  11 (18.0%) |
| **Language (n, %)**  English  Afrikaans  Tshwana  Zulu | 20 (100%)  N/A  N/A  N/A | 2 (10.0%)  4 (20.0%)  7 (35.0%)  7 (35.0%) | 2 (10.0%)  3 (15.0%)  8 (40.0%)  7 (35.0%) | 24 (40.0%)  7 (11.7%)  15 (25.0%)  14 (23.3%) | 28 (45.9%)  6 (9.8%)  15 (24.6%)  12 (19.7%) |

**Supplementary Table 2.** Illogical responses produced in each wave

|  | **Pilot (n=20)** | **Waves 1- 3 (n=61)** |
| --- | --- | --- |
| **Number of illogical responses in one or more dimensions^[[1]](#footnote-1)^** | 14 (70%) | 15 (24.59%) |
| **Percentage of responses (15 per participant) with extreme level ratings^[[2]](#footnote-2)^** | 90 (30%) | 23 (2.5%) |
| **Number of illogical or extreme anchoring responses^[[3]](#footnote-3)^** | 10 (50%) | 7 (11.48%) |

1. We define “illogical” as an objectively worse level having a rating that is higher to the rating of a better level. Such responses are considered to occur for three possible reasons: (1) a misunderstanding of the task, (2) a different understanding of the severity descriptors used (e.g. a respondent may genuinely feel that “extreme” is less problematic than “severe”), or (3) an error. [↑](#footnote-ref-1)
2. “Extreme” level ratings are defined as those with a value of 100 or zero. Such responses are not illogical, however, they are considered to be outliers that may indicate a misunderstanding of the task, or the result of the respondent wanting to complete the task quickly. [↑](#footnote-ref-2)
3. In this context an “illogical” is when a respondent rates death and full health as the same. An “extreme” responses is where respondents put the ‘pits state’ (i.e. 55555) above 0.9. These “extreme” responses are not “illogical”, however, they are considered to be outliers that may indicate a misunderstanding of the task, or the result of the respondent wanting to complete the task quickly. [↑](#footnote-ref-3)
